# Leukocyte DNA Methylation Signature Differentiates Pancreatic Cancer Patients from Healthy Controls

**DOI:** 10.1371/journal.pone.0018223

**Published:** 2011-03-24

**Authors:** Katrina S. Pedersen, William R. Bamlet, Ann L. Oberg, Mariza de Andrade, Martha E. Matsumoto, Hui Tang, Stephen N. Thibodeau, Gloria M. Petersen, Liang Wang

**Affiliations:** 1 Department of Internal Medicine, Mayo Clinic College of Medicine, Rochester, Minnesota, United States of America; 2 Department of Health Sciences Research, Mayo Clinic College of Medicine, Rochester, Minnesota, United States of America; 3 Department of Laboratory Medicine and Pathology, Mayo Clinic College of Medicine, Rochester, Minnesota, United States of America; Technische Universität München, Germany

## Abstract

Pancreatic adenocarcinoma (PaC) is one of most difficult tumors to treat. Much of this is attributed to the late diagnosis. To identify biomarkers for early detection, we examined DNA methylation differences in leukocyte DNA between PaC cases and controls in a two-phase study. In phase I, we measured methylation levels at 1,505 CpG sites in treatment-naïve leukocyte DNA from 132 never-smoker PaC patients and 60 never-smoker healthy controls. We found significant differences in 110 CpG sites (false discovery rate <0.05). In phase II, we tested and validated 88 of 96 phase I selected CpG sites in 240 PaC cases and 240 matched controls (p≤0.05). Using penalized logistic regression, we built a prediction model consisting of five CpG sites (IL10_P348, LCN2_P86, ZAP70_P220, AIM2_P624, TAL1_P817) that discriminated PaC patients from controls (C-statistic = 0.85 in phase I; 0.76 in phase II). Interestingly, one CpG site (LCN2_P86) alone could discriminate resectable patients from controls (C-statistic  = 0.78 in phase I; 0.74 in phase II). We also performed methylation quantitative trait loci (methQTL) analysis and identified three CpG sites (AGXT_P180_F, ALOX12_E85_R, JAK3_P1075_R) where the methylation levels were significantly associated with single nucleotide polymorphisms (SNPs) (false discovery rate <0.05). Our results demonstrate that epigenetic variation in easily obtainable leukocyte DNA, manifested by reproducible methylation differences, may be used to detect PaC patients. The methylation differences at certain CpG sites are partially attributable to genetic variation. This study strongly supports future epigenome-wide association study using leukocyte DNA for biomarker discovery in human diseases.

## Introduction

Pancreatic cancer (PaC) is the 10^th^ most common tumor type for men and women in yearly incidence in the United States and the fourth leading cause of cancer mortality [1]. PaC is associated with a very poor prognosis as it remains one of the most difficult tumors to treat. Much of this may be attributed to the late stage at which cancer is usually detected. Between 1999 and 2006, only 8% of patients were diagnosed, often by incidental finding on radiologic imaging, at a localized stage where immediate surgical resection and subsequent cure could be considered [2]. Currently, there are no recommended screening measures [3]. With few to no characteristic symptoms and insensitive methods for early detection, curative intervention is rare.

Epigenetics plays an important role in disease development because it unites nuclear reprogramming during development, environmentally induced changes on the body, and the ability of cells to respond appropriately to external stimuli [4]. Epigenetic variations are heritable changes in gene expression that occur in the absence of a change in the DNA sequence itself. DNA methylation, histone modification and microRNA regulation can alter the genes' expression profiles. These expression alterations often lead to the aberrant growth patterns of neoplastic cells [4].

Numerous studies point to findings that a DNA methylation profile is profoundly altered in human cancers, where global loss of DNA methylation and promoter hypermethylation are both reported [5], [6]. For PaC, previous studies have identified a panel of genes that are aberrantly methylated and silenced in tumor tissues, including *ppENK*, *SPARC*, *TFPI2*, *FOXE1*, *NPX2*, *TSLC1*, *p16*, *p14*, *p57*, and *CCND2* [7]–[12]. These genes are heavily methylated in pancreatic tumor tissues and are rarely methylated in nonneoplastic pancreas tissues [13]. A recent study reported that the expression of 58 genes was regulated by differential methylation. Additionally, 10 methylation markers were associated with altered expression of genes critical to *gemcitabine* responsiveness [14]. However, these studies have been performed using either *in vitro* systems, such as cell lines, or *in vivo* with pancreatic juice samples and primary tumor tissues, which are difficult to acquire for cancer screening due to the invasive nature of those sampling procedures [13]. Because of the risks, costs, and difficulty in obtaining pancreatic secretions and tissues for early diagnosis, a minimally invasive technique such as sampling blood is a more feasible approach for screening.

Differential methylation between cancer patients and normal controls has been reported in peripheral blood DNAs, although little is known for PaC [15]–[18]. Moore *et al.* [16] reported an association of leukocyte DNA hypomethylation with bladder cancer, independent of smoking and the other assessed risk factors. Widschwendter *et al.* [18] examined locus-specific methylation and found that particular DNA methylation patterns in peripheral blood may serve as surrogate markers for breast cancer. In a recent small cell lung cancer study, methylation profiling analysis in leukocyte DNA identified two CpG sites that jointly discriminated cancer patients from non-cancer controls [17]. These results demonstrated that methylation status in leukocyte DNA specimens may provide a useful biomarker for potential early detection and differential diagnosis. To identify methylation markers for clinical applications, we examined methylation profiles in a two-phase case-control study, which included candidate marker discovery and validation, as well as building of prediction models.

## Materials and Methods

### Ethics Statement

All subjects provided written informed consent; and the study was approved by the Mayo Clinic IRB.

### Study population

PaC index cases were adult patients with a histologically confirmed primary adenocarcinoma of the pancreas seen at Mayo Clinic between October 1, 2000 and June 1, 2006. Eligible Mayo pancreatic adenocarcinoma cases were identified through an ultra-rapid patient identification system and recruited into a prospective research registry. Study coordinators identified potential patients from the electronic patient scheduling system and daily pathology reports. All eligible patients were contacted either in the clinic at the time of their appointment, or later by mail or phone if clinic contact was not possible. If contacted at the clinic, a study coordinator obtained informed consent, arranged a venipuncture for 40 mL of blood prior to start of treatment (whenever possible), and asked the participant to complete the study questionnaire. If mail contact was required (approximately 28% of the cases were approached by mail), the study coordinator mailed an invitation letter to the patient's home address. A follow-up telephone call was made if the sample or forms were not received after 1 month. Approximately 74% of all eligible patients were enrolled into the registry. From the registry, we selected 132 never-smoker patients in phase I and 240 patients in phase II with equal representation of sex, smoking status (smoker/nonsmoker) and stage of PaC (resectable, locally advanced and metastatic).

The healthy Caucasian controls were selected from a Mayo Clinic–based research registry of primary care control patients having routine check-up visits (general medical exam) between May 1, 2004 and August 31, 2006. Controls were frequency-matched to cases on age (±5 years), sex, and state/region of residence distribution of the cases. Controls had no previous diagnosis of cancer (except non–melanoma skin cancer) at the time of enrollment. Prior to their appointment, potential controls were mailed an information brochure describing the study and a letter of invitation. On the day of the appointment, a study assistant approached the subject, confirmed eligibility criteria, and obtained informed consent. Each participant completed study questionnaires (which included a self-report of height, weight, and diabetes status) and provided 30 mL of research blood sample. Approximately 70% of all approached controls participated in this study. From this registry we selected 60 never smoker controls for phase I and 240 controls (half are never smokers) for phase II.

### DNA modification by sodium bisulfite

We extracted DNA from 5 ml of whole blood utilizing an AutoGen FlexStar (AutoGen, Inc., MA) and modified the genomic DNA specimens using the EZ DNA Methylation kit from Zymo Research Corporation (Orange, CA) that combined bisulfite conversion and DNA cleaning. The kit is based on the three-step reaction that takes place between cytosine and sodium bisulfite where cytosine is converted into uracil. We used 1 µg of genomic DNA from peripheral blood DNA for the modification per manufacturer recommendation. Treated DNA specimens were stored at −20°C and were assayed within two weeks.

### DNA methylation profiling analysis

The Illumina (San Diego, CA) GoldenGate methylation Beadchip (cancer panel) and Illumina custom VeraCode methylation assay were utilized for phase I and phase II, respectively, following the manufacturer's procedure. We imaged the arrays using a BeadArray Reader scanner (Illumina, Inc.). The proportion methylated (β-value) at each CpG site was calculated using BeadStudio Software (Illumina, Inc.) after subtracting background intensity, which was computed from negative controls, from each analytical data point. The β-value represented relative ratio of fluorescent signals between the M (methylated) allele and M+U (unmethylated) alleles. This value ranges continuously from 0 (unmethylated) to 1 (fully methylated).

### Differential methylation analysis

Due to non-Gaussian distribution of the CpG methylation values, we used Wilcoxon Rank Sum tests to examine differences in median β-values between cases and controls in both phase I and phase II. To correct for multiple testing in phase I, we used q-values to represent the false discovery rate (FDR) [19]. The CpGs with a FDR q-value ≤0.05 level were considered significant. These CpGs were then candidates for phase II validation, where a p-value ≤0.05 was considered significant. Bland-Altman plots and Spearman correlation coefficient were used to evaluate agreement between the two methylation assays in the 40 subjects assayed in both phase I and phase II. The Bland-Altman plots allow evaluation of assay disagreement as a function of level of methylation [20].

### methQTL analysis

Using PLINK (http://pngu.mgh.harvard.edu/~purcell/plink/) for this study, we performed a quantitative trait association analysis by treating each CpG β-value as the phenotype (quantitative trait), and the SNP genotypes derived from a previous genome-wide association study (GWAS) as the predictor [21]. The FDR was calculated based on the p-values from the quantitative trait association Wald test. CpG-SNP pairs with an FDR ≤0.05 were considered significant (i.e. evidence of a SNP - CpG methylation association). Because age, sex, smoking status and study phase may affect methylation levels, the methQTL analysis adjusted for these variables. Results from this analysis were used to evaluate the genetic effect on CpG methylation.

### Prediction model building

To develop prediction models, we utilized likelihood cross-validated penalized logistic regression models which implemented either an L1 penalty (Lasso) [22] or an L2 penalty (Ridge) using the R package ‘penalized’ [23]. A Lasso model (or L1 penalty) was utilized in the phase I testing study because of its desirable feature for model selection, which has a minimal effect on associated CpG coefficients while setting the unassociated CpGs' coefficients to zero. A Ridge regression model (or L2 penalty) that shrinks all coefficients to small values but not zeros was also considered for model building. The variable selection process is governed by a parameter that forces all coefficients to be shrunk near zero initially, then is gradually released to reduce the amount of shrinkage. The optimal value of this parameter is determined via cross validation. The Ridge model results were also compared to results from the Lasso model to hone the final model.

The final model identified through the penalized approaches was then fit as a generalized linear model (logistic regression) using the R package ‘glm’, in order to estimate the area under (AUC) the receiver operating characteristic (ROC) curve for each model. Models were fitted in both the testing set (phase I) and the validation set (phase II) separately with AUC reported for each model. Based on each fitted model, the probability of each individual being a case (control) was calculated. If the probability of being a case was greater than 0.50 the individual would have been classified as a case. The sensitivity is defined as the percent of cases correctly classified as cases using the model. The specificity is the percent of controls correctly classified as controls using the model.

In addition to the unadjusted model (only the CpGs), two more models were fitted, one that considered age, sex and first degree family history as covariates and another that also considered ABO blood type (‘O’ vs ‘non-O’) as an additional covariate. ABO blood types were derived for a subset of patients which had GWAS genotype information [21] available. The phase II models were fit two ways. First, coefficients from phase I were held fixed and discrimination assessed. Second, since the assay platform changed from phase I to phase II, the models were fit allowing the coefficients to be re-estimated.

## Results

### Identification of differentially methylated CpG sites in phase I

For phase I we examined 132 never-smoker patients with PaC and 60 never-smoker healthy controls. Due to chemo- or radiation therapy before blood was drawn, 13 patients were excluded from this analysis. We evaluated the methylation status (β values) of 1,505 CpG sites from leukocyte DNAs in the remaining 119 cases and 60 controls (**Table 1**). Because significant methylation differences on the X chromosome exist between males and females, we analyzed CpG sites on autosomes and sex chromosome separately. These analyses identified significant differences between PaC patients and controls at 110 CpG sites in 92 independent genes (FDR ≤0.05). 109 of the 110 significant CpG sites were located on autosomes. **Table 2** lists the 10 most significant CpG sites in the phase I study.

**Table 1:** Subject demographics for Phases I and II.

	Phase I			Phase II		
Variable	Controls (N = 60)	Cases (N = 119)	p-value	Controls (N = 215)	Cases (N = 173)	p-value
Age			1.00			1.00
≤49	3(5%)	5(4%)		20(9%)	15(9%)	
50–54	4(7%)	8(7%)		14(7%)	10(6%)	
55–59	7(12%)	12(10%)		28(13%)	21(12%)	
60–64	7(12%)	12(10%)		33(15%)	26(15%)	
65–69	12(20%)	25(21%)		39(18%)	33(19%)	
70–74	11(18%)	22(18%)		32(15%)	22(13%)	
75–79	11(18%)	22(18%)		29(13%)	29(17%)	
80–84	3(5%)	8(7%)		16(7%)	14(8%)	
≥85	2(3%)	5(4%)		4(2%)	3(2%)	
Sex			0.87			0.90
Female	31(52%)	60(50%)		108(50%)	88(51%)	
Male	29(48%)	59(50%)		107(50%)	85(49%)	
Family History of Pancreas Cancer (1^st^ degree)			0.046			0.06
No	58(97%)	104(87%)		196(91%)	147(85%)	
Yes	2(3%)	15(13%)		19(9%)	26(15%)	
Smoking Status			-			0.90
Never Smokers	60(100%)	119(100%)		97(45%)	77(45%)	
Ever Smokers	0	0		118(55%)	96(55%)	
Stage of Pancreas Cancer			-			-
Resectable		31(26%)			58(34%)	
Locally Advanced		45(38%)			59(34%)	
Metastatic		43(36%)			56(32%)	
GWAS genotyping			<0.001			0.028
No		32(27%)		106(49%)	66(38%)	
Yes	26(43%)	87(73%)		109(51%)	107(62%)	

**Table 2:** Top 10 most differentially methylated CpG sites in phase I and validation in phase II.

	Phase I				Phase II			
Illumina ID	Median β Control	Median β Case	Difference (case-control)	p value	Median β Control	Median β Case	Difference (case-control)	p value
ITK_P114_F	0.8337	0.9006	0.0669	< 1E-10	0.846	0.8898	0.0438	< 1E-10
LCN2_P86_R	0.5608	0.4398	−0.121	2.00E-10	0.591	0.4993	−0.0917	< 1E-10
ITK_E166_R	0.8859	0.9414	0.0555	5.00E-10	0.8885	0.9299	0.0414	< 1E-10
PECAM1_E32_R	0.2319	0.1566	−0.0753	1.60E-09	0.2851	0.2211	−0.064	< 1E-10
LMO2_E148_F	0.3885	0.2704	−0.1181	2.30E-09	0.4969	0.3904	−0.1065	< 1E-10
IL10_P348_F	0.6026	0.4597	−0.1429	2.50E-09	0.7191	0.6382	−0.0809	< 1E-10
LCK_E28_F	0.8114	0.8684	0.057	3.60E-09	0.8593	0.8999	0.0406	< 1E-10
RUNX3_P247_F	0.7837	0.8672	0.0835	5.90E-09	0.7528	0.841	0.0882	< 1E-10
LMO2_P794_R	0.3143	0.2027	−0.1116	1.02E-08	0.3754	0.3027	−0.0727	6.00E-10
MMP14_P13_F	0.4721	0.3472	−0.1249	2.27E-08	0.5694	0.4807	−0.0887	< 1E-10

To evaluate for potential gender differences, we compared the 1,421 autosomal CpG sites between males and females. We observed significant differences at 71 CpG sites when combining cases and controls. When analyzed these CpG sites in cases and controls separately, we observed differences in 44 CpG sites for PaC patients and 6 CpG sites for controls (**Table S1**). To evaluate possible methylation changes during tumor progression, we examined the methylation differences among three stages of PaC within this patient population including 31 resectable, 45 locally advanced, and 43 metastatic cases. Although nine CpG sites showed a trend in association with clinical stage (p<0.01) (**Table 3**), the data analysis did not reveal significant difference among the three stages (all CpG sites with FDR >0.05).

**Table 3:** Top 10 most differentially methylated CpG sites among 3 clinical stages.

Illumina ID	Gene Name	Mean β values			p value	FDR
		Resectable	Locally Advanced	Metastatic		
ZMYND10_P329_F	ZMYND10	0.045	0.032	0.019	0.001	0.722
EPO_P162_R	EPO	0.077	0.046	0.068	0.001	0.722
SCGB3A1_P103_R	SCGB3A1	0.004	0.020	0.004	0.002	0.722
MEST_P4_F	MEST	0.042	0.029	0.061	0.002	0.722
PWCR1_P357_F	PWCR1	0.917	0.920	0.890	0.003	0.722
NTRK3_P636_R	NTRK3	0.009	0.009	0.004	0.003	0.722
TIE1_E66_R	TIE1	0.203	0.161	0.153	0.006	1.000
HLA_DPA1_P205_R	HLA	0.065	0.041	0.052	0.007	1.000
EDNRB_P148_R	EDNRB	0.995	0.995	0.995	0.009	1.000
COL1A2_P48_R	COL1A2	0.033	0.023	0.028	0.011	1.000

### Validation of selected CpG sites in phase II

**Figure 1:**
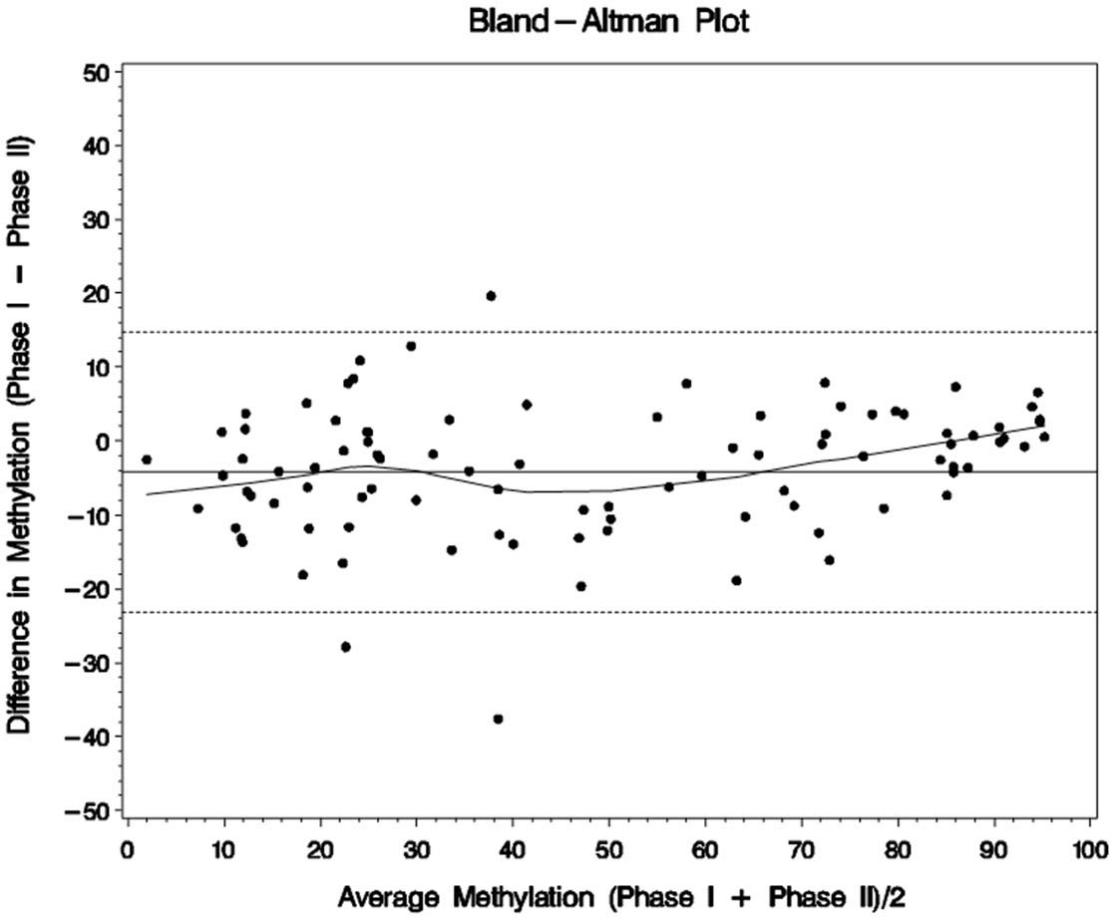
Methylation level agreement between phase I and phase II. Representative Bland-Altman graph in one subject demonstrates good agreement between phase I and phase II data in most 96 CpG sites. Each dot represents one CpG site. Mean methylation level for each CpG site (from 0 to 100%) is shown in x-axis. Methylation level difference for each CpG site between phase I and phase II is shown in y-axis. The dashed lines indicate 95% confidence interval for the difference between the two assays and the solid line indicates the average differences between the two assays.

To validate the differentially methylated CpG sites identified in phase I within a larger number of patients and a broader range of demographic characteristics, we designed a custom VeraCode methylation assay (Illumina, Inc.) and examined 96 of the 110 significant CpG sites in 240 PaC cases and 240 matched controls. The 96 CpG sites were selected according to median β differences between cases and controls. For CpG sites with similar median β differences, the CpG sites with smaller FDR were selected. Among the 480 subjects, 40 phase I subjects (20 cases and 20 controls) were included in order to compare the degree of agreement between the two methylation assays. Bland Altman plots [20] showed little mean shift and constant variation of differences over the range of values (**Figure 1**), demonstrating reasonable agreement between the two assays. The two assays were significantly correlated as expected among all 96 CpG sites. The median Spearman correlation coefficient r was 0.94 (range from 0.84 to 0.96).

**Figure 2:**
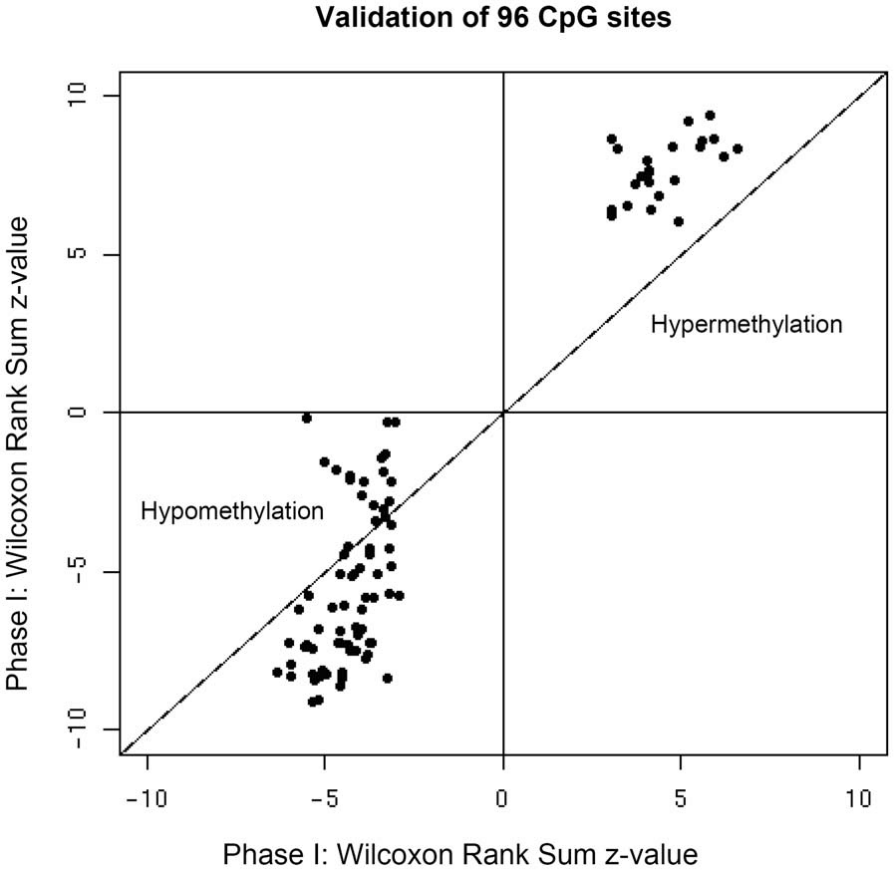
Validation of 96 selected CpG sites. Scatter plot shows reproducible methylation differences between phase I and phase II. Wilcoxon Rank Sum z-values were plotted on x-axis (phase I) and y-axis (phase II). 88 of the 96 CpG sites were validated by p value (<0.05) and direction (hyper/hypo-methylation). Although 8 CpG sites were not statistically significant, the trends in both phases are all the same.

Among the 220 PaC patients who were unique to phase II, 47 patients had been treated before blood was drawn. We compared the methylation levels between these 47 treated cases and 173 never-treated cases to evaluate the effect of treatment on the methylation status of these selected CpG sites. Two CpG sites (TAL1_P817_F and CSF3_E242_R) showed nominal differences (p = 0.001 and 0.025, respectively), although these results could be due to chance, given the large number of comparisons. Overall, we did not observe a significant treatment effect on the methylation of these selected CpG sites. Similarly, no effect was attributable to smoking history (data not shown). Of the remaining 220 controls, five additional controls were excluded due to inadequate quality, leaving 215 controls who were unique to phase II (**Table 1**). A total of 173 never-treated cases and 215 controls were analyzed in phase II. The Wilcoxon Rank Sum Test identified a significant difference (p≤0.05) in 88 of the 96 selected CpGs. Importantly, all 88 of these validated CpG sites in phase II also showed the same direction of methylation change as phase I (**Figure 2**). Of those, 23 and 65 CpG sites demonstrated hypermethylation and hypomethylation in PaC patients, respectively. **Table 2** lists the 10 most significant CpG sites in the phase II study (**Table S2** contains statistics of the 96 CpG sites in both phases I and II).

### Genetic effect on DNA methylation

**Figure 3:**
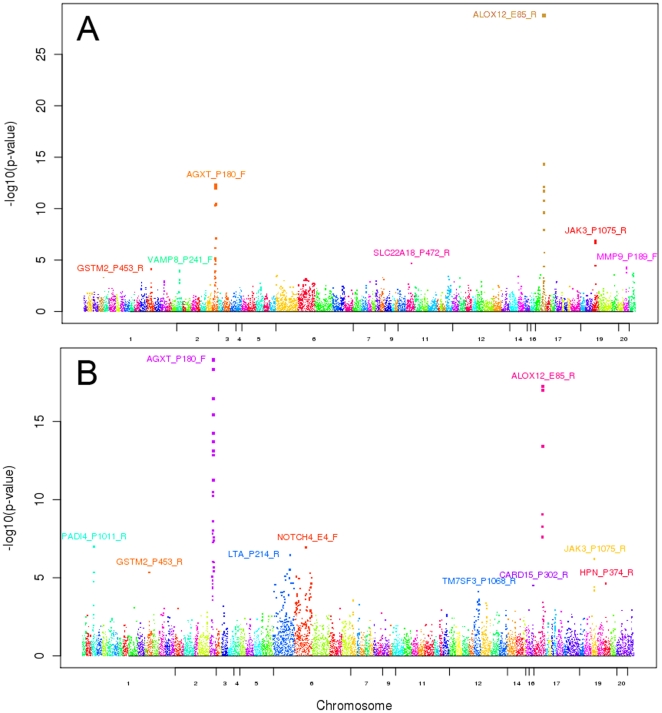
Manhattan plot for methQTL analysis for 96 CpG sites. Methylation levels for each of 96 CpG sites were regressed on copy number of minor alleles at nearby (+/−1 Mb) SNPs. The CpG sites were placed on x-axis based on their order of chromosomal location. Y-axis was –log10 of methQTL p value after adjusting for age and sex. Size of each dot was proportional to the significance of each p value. **A.** control-only methQTLs and **B.** case-only methQTLs.

To examine whether there was any genetic influence on CpG methylation, we regressed the methylation level (β value) for each of 96 phase II CpG sites on corresponding SNP genotypes within 1 MB of the target CpG site. There were 16,217 genotyped SNPs surrounding the 96 CpG sites with a minor allele frequency ≥0.1. 135 controls and 194 patients had SNP genotype data. Using an additive model and adjusting for age and sex, we identified 33 CpG-SNP pairs (methQTLs) in controls and 99 methQTLs in PaC cases with an FDR ≤0.05 (**Figure 3** **and Table S3**). Of those, 24 methQTLs were shared between both cases and controls. These shared methQTLs involved three independent CpG sites. The methQTLs with the strongest associations for the three CpG sites were ALOX12_E85_R and rs434473 (FDR = 1.66×10^−25^ in controls and 3.01×10^−14^ in patients), AGXT_P180_F and rs4675872 (FDR = 1.83×10^−9^ in controls and 3.73×10^−15^ in patients), and JAK3_P1075_R and rs7245564 (FDR = 1.89×10^−4^ in controls and 3.48×10^−4^ in patients), respectively (**Table 4**). The distances between the SNPs and target CpGs were less than 15.5 Kb for all three pairs reported here. We also performed the methQTL analysis adjusting for phases of the study and/or smoking history in addition to age and sex. These results were consistent with those when adjusting for age and sex alone (**Table S3**).

**Table 4:** Significant association between CpG sites and nearby (+/−1 Mb) SNPs.

Gene Name	Chr	CpG site		SNP		methQTL FDR*	
		Target ID	Position	SNP with best p value	Position	Controls	Cases
AGXT	2	AGXT_P180_F	241,456,655	rs4675872	241,472,126	**1.83E-09**	**3.73E-15**
ALOX12	17	ALOX12_E85_R	6,840,213	rs434473	6,845,658	**1.66E-25**	**3.01E-14**
JAK3	19	JAK3_P1075_R	17,820,875	rs7245564	17,824,878	**1.89E-04**	**3.48E-04**

*The reported FDRs were based on SNP-CpG association after adjusting age and sex. Please see Table S3 for more statistics after adjusting age, sex, phases of the study and/or smoking history.

### Building and validation of the prediction model

To build prediction models based on phase I data, we first excluded 43 of the 96 CpG sites that showed less than 5% median β differences between cases and controls or p-value ≥0.001 (FDR>0.007) in phase I. These filter criteria were set for the following technical considerations. First, CpG sites with smaller methylation differences are prone to laboratory error due to technical limitations. Second, CpG sites with less significant p-values are less likely to be replicated in a future study. Based on 53 remaining CpG sites, we first built models using L1 and L2 penalties as described in the methods using the phase I data. The best model was chosen based on criteria of ROC AUC and parsimony. This model was then tested using the phase II data without the 40 subjects assayed in both phases for the agreement study. When considering all cases and all controls, we identified a panel of five CpG sites (**Model I**: IL10_P348, LCN2_P86, ZAP70_P220, AIM2_P624, TAL1_P817) that were the first five CpGs to enter and remain in the Lasso model and also the five largest coefficients from the Ridge model. This five CpG-only model showed good discrimination between patients and controls (c-statistic  = 0.85 in phase I and 0.76 in phase II) based on the logistic regression model. When specificity was set to 0.90, the maximum sensitivity was 0.65 in phase I and 0.51 in phase II. When specificity was set to 0.70, the maximum sensitivity was 0.83 in phase I and 0.72 in phase II (see **Table S4** for full spectrum of specificity and sensitivity). When including covariates in the logistic regression model (age, sex, 1^st^ degree of family history of PaC, ABO blood type), the discrimination was improved in phase I (c-statistic  = 0.89), but decreased in phase II (c-statistic  = 0.72). When re-estimating coefficients of these covariates in phase II (re-fitting), the discrimination was improved as expected, but not dramatically (c-statistic = 0.77 for five CpGs only, 0.77 after inclusion of covariates) (**Table 5**). When including resectable patients only and all controls, we identified one CpG site (**Model II**: LCN2_P86) that appeared to discriminate for resectable disease (c-statistic  = 0.78 in phase I and 0.74 in phase II).

**Table 5:** Methylation-based predication models and Area Under the ROC Curve (AUC).

		Phase I			Phase II			Phase II –Re-fit		
Models	CpG Illumina ID	CpGs only	CpG+ Covariates*	CpG+ Covariates* +ABO**	CpGs only	CpG + Covariates*	CpG+ Covariates* +ABO**	CpGs only	CpG + Covariates*	CpG+ Covariates* +ABO**
**All Cases and All Controls**		**60 controls, 119 cases**			**215 controls, 173 cases**			**215 controls, 173 cases**		
**I**	IL10_P348 LCN2_P86 ZAP70_P220 AIM2_P624 TAL1_P817	0.85	0.86	0.89	0.76	0.75	0.72	0.77	0.77	0.77
**Resectable Cases and All Controls**		**60 controls, 31 cases**			**215 controls, 58 cases**			**215 controls, 58 cases**		
**II**	LCN2_P86	0.78	0.79	0.82	0.74	0.67	0.64	0.73	0.73	0.73

*Covariates include age, sex, 1st degree family history of PaC**.**

**ABO-blood type of O and non-O.

## Discussion

In this two phase (testing-validation) study, we examined peripheral blood DNA to determine whether differences in methylation at various CpG sites could distinguish between subjects with and without PaC. We limited our study to those patients who had never been treated for cancer (both phase I and II), and in phase I to those who had never smoked. We demonstrated highly significant hypo- or hypermethylation loci in leukocyte DNA of the patients with PaC. This was true in both phases of the study and appeared to remain significant with adjustment for smoking status and cancer treatment. Importantly, we found that the methylation differences were not significant across the various stages of the disease but significant between resectable patients and controls, suggesting that the differentially methylated CpG signatures may be useful for early diagnosis.

It is worth emphasizing that at least four (*LCN2*, *IL10*, *PECAM1* and *MMP14*) of the top ten most significantly methylated genes in this study have previously been reported to have diagnostic and/or prognostic value for PaC. For example, studies have shown that serum *IL-10* levels are significantly elevated in PaC patients [24], [25]. Patients who had higher levels of *IL-10* showed significantly worse survival compared with patients who showed lower *IL-10* levels. The elevated level of *IL-10* in serum of PaC patients is consistent with our current finding that leukocyte DNA had lower promoter CpG methylation of the gene in PaC patients than in controls (p  = 2.50×10^−9^ for phase I and p<1×10^−10^ for phase II). Interestingly, another gene, *LCN2*, is proposed as an early diagnosis biomarker for PaC [26]–[28]. One expression-based study showed that levels of five proteins, including *LCN2*, discriminated between PaC patients and matched controls up to 13 months before cancer diagnosis [26]. In this study using leukocyte DNA, we found that two CpG sites (LCN2_P86_R and LCN_P141_R) within the *LCN2* promoter region showed significantly lower methylation levels in PaC patients than in controls (p =  2.00×10^−10^ and 1.16×10^−7^ in phase I, p<1×10^−10^ and <1×10^−10^ in phase II, respectively). Based on a recent twin study, it appears that the hypomethylation is associated with increased *LCN2* expression in leukocytes [29]. Furthermore, our model algorithm ranked IL10_P348 and LCN2_P86 as the top two CpG sites selected in Model I and LCN2_P86 as the only CpG site selected in Model II. These results, therefore, strongly support that DNA methylation status in leukocytes may serve as biomarkers to assist in PaC diagnosis.

Although sequence dependent allele-specific methylation is a unique feature of imprinted genes in the human genome, the DNA modification has also been observed in non-imprinted loci. Several recent studies have demonstrated that genetic variants may have a significant effect on DNA methylation at various loci [30]–[33]. Using array-based assays, two studies have identified multiple genotype-epigenotype interactions by showing significant association between CpG methylation levels and SNP genotypes [30], [33]. Boks et al. analyzed leukocyte DNA methylation in twins and healthy controls, and identified a significant genetic influence on the methylation of multiple CpG sites [30]. Gibbs et al. surveyed 27,578 CpG sites for association with 1.63 million SNPs in four types of brain tissues [33]. For each tissue type, 4–5% of these CpGs showed significant association with at least one SNP after correction for genome-wide multiple testing. Overall, 3,619 unique CpG sites were reported to be methQTLs. Of those, 887 were cis-methQTL. Surprisingly, peak enrichment for the methQTL was only 45 bp to the CpG site in question [33]. In this study, we found that at least three (3.13%) of the 96 selected CpG sites were significantly affected by cis-acting SNPs. We also found that methQTL SNPs were generally close to the target CpG site (<15.5 Kb). These results demonstrate that CpG methylation levels at some non-imprinting loci are also highly regulated by nearby genetic variants. The methylation status in peripheral blood DNA may also serve as biomarkers for risk assessment of PaC.

While the results of this study are promising, more work is needed to determine the causes of these methylation differences and whether differential methylation could be further validated as a screening or diagnostic tool in an unselected population. At this point, it is unknown what causes the methylation differences between these patients and controls. In addition to possible genetic effect as described above, immune response of lymphocytes to cancer cells is another plausible explanation. It is also unknown whether comorbid conditions may affect the sensitivity of this test and whether precancerous lesions or benign disease, such as pancreatitis, would exhibit similar methylation patterns at these CpG sites. Nevertheless, these results may have future clinical significance. First, the highly reproducible results in the study suggest that methylation differences do exist between PaC cases and controls, which provide support in the rationale of designing methylation-based assay for early cancer detection and even risk assessment. Second, utilizing easily accessible leukocyte DNAs, rather than pancreatic tissue- or juice-based substrate, is amenable to large-scale epigenetic epidemiology applications such as epigenome-wide association study. Third, the study identified a significant role of genetics on DNA methylation at some disease-related CpG sites. It is critical to discriminate between genetic and non-genetic effects on DNA methylation. While changes in methylation as a result of a disease may be a useful marker for early diagnosis, methylation variation due to genetic influences may be a potential marker for risk assessment across individuals in larger populations. Therefore, characterization of a genetic role on DNA methylation will facilitate biomarker discovery and further stratify clinical applications for these molecular signatures.

In summary, we have confirmed reproducible methylation differences between PaC patients and controls and have identified a set of differentially methylated CpG sites that appear to be highly indicative of the presence of PaC. If further validated and improved in future studies, these results may be applied to a feasible screening regimen, either in high-risk population or even in the general population, to detect this cancer at potentially curative stages.

## Supporting Information

Table S1 Gender methylation differences on autosomes.
(XLS)

Table S2 Summary statistics (median (min, max)) of the 96 significantly differentially methylated CpG sites by phase and case/control status.
(XLS)

Table S3 methQTLs in disease-related CpG sites (controls and cases separately).
(XLS)

Table S4 Sensitivity and Specificity for the 5 CpGs only model.
(XLS)
